# CRP and leukocyte-count after lumbar spine surgery: fusion vs. nucleotomy

**DOI:** 10.3109/17453674.2011.588854

**Published:** 2011-09-02

**Authors:** Clayton N Kraft, Tobias Krüger, Jörn Westhoff, Christian Lüring, Oliver Weber, Dieter C Wirtz, Peter H Pennekamp

**Affiliations:** ^1^Department of Orthopaedics, Trauma Surgery and Hand Unit, Helios Klinikum, Krefeld; ^2^Department of Orthopaedic Surgery, University of Regensburg, Bad Abbach; ^3^Department of Orthopaedic and Trauma Surgery, Rheinische Friedrich Wilhelms University, Bonn, Germany; Correspondence: clayton.kraft@helios-kliniken.de

## Abstract

**Background:**

Despite the fact that C-reactive protein (CRP) levels and white blood cell (WBC) count are routine blood chemistry parameters for the early assessment of wound infection after surgical procedures, little is known about the natural history of their serum values after major and minimally invasive spinal procedures.

**Methods:**

Pre- and postoperative CRP serum levels and WBC count in 347 patients were retrospectively assessed after complication-free, single-level open posterior lumbar interlaminar fusion (PLIF) (n = 150) for disc degeneration and spinal stenosis and endoscopically assisted lumbar discectomy (n = 197) for herniated lumbar disc. Confounding variables such as overweight, ASA classification, arterial hypertension, diabetes mellitus, and perioperative antibiotics were recorded to evaluate their influence on the kinetics of CRP values and WBC count postoperatively.

**Results:**

In both procedures, CRP peaked 2–3 days after surgery. The maximum CRP level was significantly higher after fusion: mean 127 (SD 57) (p < 0.001). A rapid fall in CRP within 4–6 days was observed for both groups, with almost normal values being reached after 14 days. Only BMI > 25 and long duration of surgery were associated with higher peak CRP values. WBC count did not show a typical and therefore interpretable profile.

**Conclusion:**

CRP is a predictable and responsive serum parameter in postoperative monitoring of inflammatory responses in patients undergoing spine surgery, whereas WBC kinetics is unspecific. We suggest that CRP could be measured on the day before surgery, on day 2 or 3 after surgery, and also between days 4 and 6, to aid in early detection of infectious complications.

In spinal surgery, the rate of postoperative deep wound infection depends on the type of surgery and on patients' premorbidity, and ranges from 0.7% for simple nucleotomy to over 3.5% for fusion with instrumentation, and can reach 20% in patients with metastatic disease (Wimmer et al. 1998, Weinstein et al. 2000). Early detection of postoperative infection, especially in spine surgery, can be difficult. Numerous plasma values have been used in monitoring of infection-related responses after spine surgery. These plasma determinations range from simple and inexpensive tests such as erythrocyte sedimentation rate (ESR) (Thelander and Larsson 1992, Meyer et al. 1995) to the complex determination of soluble biomarkers of inflammatory response such as quantification of cytokines (Huang et al. 2005). The value of ESR as an early marker of infection has been disputed (Larsson et al 1990, Meyer et al. 1995, Khan et al. 2006) and the latter methods are complicated and difficult to interpret, and are also very expensive.

The acute-phase-related C-reactive protein (CRP) is a feasible parameter for detection and monitoring of postoperative wound infections and systemic inflammatory response syndromes (Foglar and Lindsey 1998, Neumaier et al. 2006, Takahashi et al. 2006). Serial CRP measurements are useful not only as a diagnostic tool for infection, but also for monitoring the effect of treatment and for the early detection of relapse (Larsson et al 1990, Codine et al. 2005, Mok et al. 2008). As with other orthopedic surgical procedures, surgery of the spine induces a temporary rise in CRP (Garnavos et al. 2005, Neumaier et al. 2006, Mok et al. 2008). One prerequisite for the use of CRP as a diagnostic aid after spine surgery in the early detection of postoperative infection is an understanding of the natural CRP response induced by the procedure. To establish this baseline, we examined CRP levels and white blood cell (WBC) count after 2 types of surgical procedures of the lumbar spine. In addition, we assessed the degree to which postoperative CRP kinetics is influenced by patient comorbidity and by perioperative administration of prophylactic antibiotics.

## Patients and methods

We retrospectively assessed 1,320 patients who underwent surgery of the spine at our university hospital during 2001–2004, and for this study selected patients (aged over 18 years) who received single-level fusion for lumbar spinal stenosis (fusion) and single-level nucleotomy for herniated lumbar disc (nucleotomy). Only patients with normal levels of CRP before surgery were included. Exclusion criteria were staged procedures, surgery for infection, postoperative hematoma, history of autoimmune or inflammatory disorders, hepatitis or liver disease, tumor or cancer, or infectious complications after surgery. 347 patients (150 fusion, 197 nucleotomy) met the inclusion criteria and were included in the study. Comorbidity information and the ASA score were obtained from the preoperative history, records of physical examination, and anesthesia records. We also recorded region of lumbar surgery, type of procedure, operative time from skin incision to closure, estimated blood loss, and whether or not prophylactic antibiotics were administered.

### Surgery

All procedures were performed under general anesthesia. Patients receiving antibiotics were treated with cefazolin (1 g) intravenously within 1 h of skin incision, and for those with nucleotomy in the form of a single-shot administration; in those with fusion surgery, administration of antibiotics was continued every 8 h over 24 h postoperatively. In the presence of a penicillin allergy, clindamycin was used instead of cefazolin.

Surgical management of herniated lumbar disc was performed by a minimally invasive endoscopically-assisted technique using the endospine system (Karl Storz, Tuttlingen, Germany) as described by Destandau (2004). The steps of the procedure and the iatrogenic trauma to paraspinal muscle are comparable to the widely used method of limited lumbar microdiscectomy using an operating microscope. Patients who received single-level fusion for spinal stenosis were treated using a 360° posterior lumbar interlaminar fusion (PLIF) technique. After sufficient spinal canal decompression, 4 transpedicular screws (XIA Spine System; Stryker, Kalamazoo, MI) were placed under fluoroscopic control. After complete removal of the disc space and freshening of the adjacent endplates, 2 polyetheretherketone (PEEK) cages (PLIF Spine System, Stryker) filled with bone graft harvested from the removed spinous process were fitted. Over the rods, compression was applied to the anterior cages and the rods were then firmly fixed to screws to achieve a stable 360° instrumentation.

In all patients, blood specimens were obtained on the day before surgery and on the day after surgery. Four further specimens were taken on days 2 or 3, 4–6, 7–9, and 10–14 after the procedure. The samples were obtained at approximately the same time each morning. Serum CRP was quantified by the CardioPhase high- sensitivity C-reactive protein (CCRP) method with heterogeneous immunoassay (CardioPhase high-sensitivity CRP, Siemens Healthcare Diagnostics, Marburg, Germany). White blood cell count was obtained using flow cytometry (Siemens Healthcare Diagnostics).

### Statistics

Continuous variables such as age, body weight, duration of surgery, and the peak values of the inflammatory parameters are given as mean (SD). The difference between the two treatment modalities regarding CRP and WBC were assessed using Wilcoxon rank-sum tests. Categorical variables were presented in frequencies (relative frequencies), and the heterogeneities between the groups were tested using Chi-square test or Fisher's exact test, as appropriate, if the expected frequencies were less than 5. Probability values were not adjusted for the multiple testing. To estimate the effect of treatment modality on the peak values of inflammatory parameters with adjustment for the potential confounding factors, multifactorial linear regression models were used. The statistically significant factors (p < 0.05) regarding general characteristics in the univariate analyses (cf. Table 1) were included in the model, in addition to the baseline values of the same parameters. The analyses were performed using SAS software version 8.2 (SAS Institute Inc., Cary, NC).

**Table 1.** General patient characteristics (n = 347), medical history, and comorbidities

General characteristics	Nucleotomy	Fusion surgery	p-value
	(n = 197)	(n = 150)	
Age, years	47 (15)	55 (15)	< 0.001
Gender, male	99 (50%)	76 (51%)	1.0
Body weight, kg	79.3 (18)	79.3 (15)	0.9
BMI > 25	39 (20%)	44 (29%)	0.04
Duration of surgery, h	0.86 (0.65)	2.32 (1.16)	< 0.001
Antibiotic treatment			
Preoperative administration (24 h)	0 (0%)	4 (2.7%)	0.03
Single-shot intraoperatively	65 (33%)	120 (80%)	< 0.001
Postoperative administration (24 h)	45 (23%)	117 (78%)	< 0.001
Comorbidities and prior surgery			
Diabetes mellitus	9 (4.6%)	8 (5.3%)	0.80
Arterial hypertension	24 (12%)	49 (33%)	< 0.001
Coronary heart disease	4 (2.0%)	10 (6.7%)	0.05
History of thrombosis	2 (1.0%)	2 (1.3%)	1.0
History of hip surgery	2 (1.0%)	2 (1.3%)	1.0
History of knee surgery	6 (3.1%)	3 (2.0%)	0.7
History of abdominal surgery	6 (3.1%)	2 (1.3%)	0.5
History of nucleotomy	17 (8.6%)	12 (8.0%)	1.0
ASA classification			
I	72 (37%)	21 (14%)	< 0.001
II	21 (11%)	30 (20%)	0.05
III	104 (53%)	98 (65%)	0.02
IV	0 (0%)	1 (0.7%)	< 0.001
V	0 (0%)	0 (0%)	NA

The p-values are means from Chi-square or Fisher's exact tests. Age, body weight, and duration of surgery are given as mean (SD). The p-values are derived from Wilcoxon rank-sum tests. The heterogeneities of sex and obesity were tested with Chi-square tests. NA: Not applicable .

## Results

347 patients met the inclusion criteria and were included in the study (Table 1).

### Kinetics of CRP

**Figure 1.**
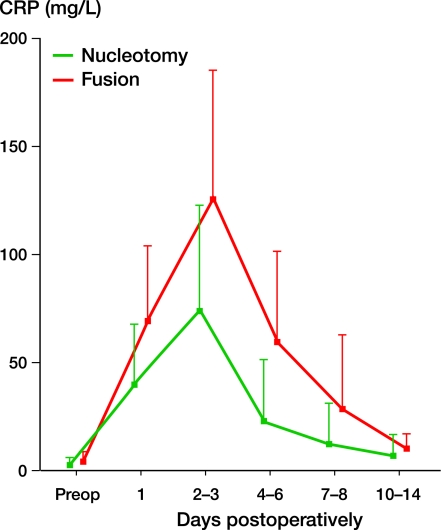
Kinetics of postoperative CRP levels (mg/L): fusion (n = 150) vs. nucleotomy (n = 197). Values are mean and standard deviation.

Preoperative baseline values of CRP were normal: 3.0 (SD 3.1) mg/L for patients treated with nucleotomy (n = 197) and 4.3 (SD 4.6) mg/L for those treated with fusion (n = 150). For both types of surgery a characteristic postoperative CRP pattern was observed, although the maximum amplitude varied between the procedures. In 90% of patients who underwent nucleotomy (178/197) and in 81% of patients with fusion (122/150), peak CRP values were reached between days 2 and 3 postoperatively (p = 0.02, Fisher's exact test). While maximum CRP values after nucleotomy increased approximately 25 fold to 75 (SD 48) mg/L, the increase was higher after fusion, with an approximately 30-fold increase to 127 (SD 58) mg/L relative to baseline values (Table 2). This discrepancy in peak CRP levels was statistically significant (p = 0.007). Peak CRP values after both procedures were followed by a rapid decrease in all patients. At the 4–6-day control, the mean value was 23 (SD 28) mg/L after nucleotomy and 60 (SD 42) mg/L after fusion. At the 10–14-day control, the mean values were 7.1 (SD 6.8) mg/L after nucleotomy and 10 (SD 6.8) mg/L after fusion, thus approaching normal values (< 5 mg/L) (Figure 1).

**Table 2.** Mean profiles of CRP and leukocyte values preoperatively and postoperatively

	Nucleotomy	Fusion surgery	p-value
	(n = 197)	(n = 150)	
*CRP (mg/L)*			
Preoperatively	3.0 (3.1)	4.3 (4.6)	< 0.001
Day 1	41 (27)	70 (34)	< 0.001
Day 2–3	75 (48)	127 (58)	0.007
Day 4–6	23 (29)	60 (42)	< 0.001
Day 7–9	13 (19)	29 (34)	< 0.001
Day 10–14	7.1 (9.6)	10 (6.8)	< 0.001
*Leukocytes (G/L)*			
Preoperatively	8.5 (2.9)	7.8 (2.8)	0.02
Day 1	9.3 (3.2)	10.1 (3.0)	0.01
Day 2–3	9.9 (4.4)	9.5 (2.7)	0.6
Day 4–6	7.2 (2.5)	7.3 (2.7)	0.8
Day 7–9	7.3 (2.4)	8.1 (2.7)	0.08
Day 10–14	8.1 (2.5)	8.0 (3.0)	0.6

The inflammatory parameters are summarized as mean (standard deviation). The p-values were derived from Wilcoxon rank-sum tests.

### Leukocyte kinetics

**Figure 2.**
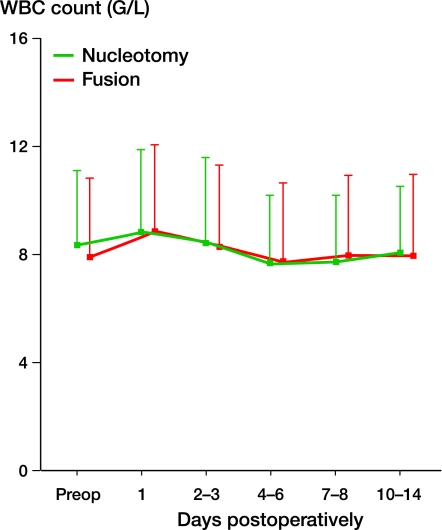
Kinetics of postoperative white blood cell count (g/L): fusion (n = 150) vs. nucleotomy (n = 197). Values are mean and standard deviation.

Preoperative baseline values of WBC count were normal and approximately the same (8.5 (SD 2.9) g/L) for patients treated with nucleotomy or fusion. For both types of surgery, leukocyte count had a characteristic pattern: the maximum amplitude varied statistically significantly between the two procedures only on the first postoperative day (Table 2). Maximum leukocyte counts were back to or below preoperative values between 4–6 days after surgery (Figure 2).

### Influence of comorbidities and surgery time

Using multiple regression analysis, we assessed whether there might be any correlation between peak inflammatory parameters and comorbidities. In neither of the cohorts did diabetes mellitus, arterial hypertension, compensated heart insufficiency, nor a history of prior lumbar nucleotomy in another segment have any influence on CRP or leukocyte kinetics postoperatively compared to patients without these comorbidities (Table 3). Surprisingly, ASA classification as a parameter of patients' overall health status and history of spine surgery were not found to affect postoperative peak CRP values or leukocyte values (or their normalization). In contrast, duration of surgery had a statistically significant influence on postoperative peak values of CRP and leukocytes: the longer the surgery, the higher the peak levels (p = 0.04). While we found an association between obesity (BMI > 25) and postoperative peak CRP levels, we found no association between obesity and leukocyte values (Table 3).

**Table 3.** Multiple regression analysis of peak values of CRP and leukocyte profiles for all patients (n = 347)

	Peak CRP	Peak leukocytes
Multiple regression	0.28	0.62
F-value	23	99
p-value	< 0.001	< 0.001
Intercept		
Coefficient (SE)	4.89 (8.6)	1.56 (0.50)
p-value	0.6	0.002
OP procedure		
Coefficient (SE)	30 (7.3)	0.35 (0.32)
p-value	< 0.001	0.27
Surgical duration		
Coefficient (SE)	5.9 (2.9)	0.50 (0.13)
p-value	0.04	< 0.001
Baseline value		
Coefficient (SE)	2.5 (0.7)	0.81 (0.04)
p-value	0.001	< 0.001
Perioperative AB use		
Coefficient (SE)	–0.83 (5.7)	0.02 (0.25)
p-value	0.9	0.9
Obesity		
Coefficient (SE)	14 (5.9)	–0.06 (0.25)
p-value	0.02	0.8

SE: standard error. Multifactorial linear regression models were used

### Influence of antibiotics

Regression analysis showed that administration of antibiotics did not have any influence on peak serum CRP or leukocyte values. We did not find any delay in recovery of these values after antibiotic treatment.

## Discussion

Analogous to what has been found for other surgical procedures, we observed a rise in CRP on day 1 after elective spine surgery and peaks in CRP between days 2 and 3 after intervention, indicating a major inflammatory response. The intensity of this operative peak was individual and it was of no prognostic relevance. Even so, while peak values varied significantly between individuals, there was a remarkable resemblance in CRP profiles between patients subjected to the same procedure. In a fairly small patient series (n = 149) with varying types of spinal procedures, Mok et al. 2008) found that postoperative CRP kinetics in spine surgery appeared to be similar regardless of operation, magnitude, or region. We share their opinion that peak values serve only as a reference value for the future time course. Independently of the type of spine surgery, CRP levels then fall rapidly. If this is not the case and CRP levels are persistently high at the 5–7-day control, it is indicative of an infectious complication. As suggested by Thelander and Larsson 1992), a rapid decline in CRP levels after spine surgery will be interrupted by a second rise or persistent elevation if infection occurs. Of the 1,320 patients who underwent a spinal procedure in our university hospital, most of them with an infectious complication (3.1%, n = 41) showed a steady postoperative increase in CRP levels, frequently accompanied by persistent back pain and/or local signs of wound infection (data not shown).

That different surgical procedures have varying inflammatory peak responses is not new. In orthopedic surgery, maximum postoperative CRP levels depend heavily on the region and type of surgery performed (Larsson et al 1990, Thelander and Larsson 1992, Kragsbjerg et al. 1995, Niskanen et al. 1996, Neumaier et al. 2006). In general, this varying peak response depends on the amount of iatrogenic tissue injury at surgery. We found the same in our study. However, Larsson et al 1990) compared different types of orthopedic surgical procedures and could not always find a correlation between the extent of surgery and peak CRP levels postoperatively. They postulated that the increase in CRP depends not only on the amount of tissue injured but also on the type of tissue being damaged.

Numerous factors favor CRP as being a more effective laboratory test than WBC count. CRP level is stable for an individual, and has a narrow normal range. It is hardly influenced by drugs and—as we found also—it appears that prior spine surgery, common comorbidities, or pathologies (apart from liver failure) have no effect (Pepys and Hirschfeld 2003). On the other hand, determination of WBC count is considerably less expensive. In instrumented spinal surgery especially, it is, however, only of interpretational value in combination with erythrocyte sedimentation rate (ESR), body temperature, and CRP levels—even in cases with proven infectious complications (Takahashi et al. 2001). Our data highlight the fact that in contrast to CRP, WBC count after complication-free spinal surgery does not show a typical (and therefore interpretable) profile, making it of secondary value only as a serum parameter for early detection of infectious complications.

Since it has been shown that there is a correlation between serum CRP levels and clinical response in patients treated with antibiotics for wound infections after spinal surgery (Khan et al. 2006), we wanted to know whether prophylactic perioperative administration of antibiotics reduces the interpretational value of postoperative CRP values. This was not the case, which is important from a clinical point of view. Administration of antibiotics over a short period of time perioperatively does not affect CRP values.

For clinical practice in patients undergoing elective spine surgery, we suggest routine measurement of serum CRP levels on the day before surgery, and on days 2–3 and 4–6 postoperatively. With no clinical findings suspect for infection a marked drop in CRP-values on the 4-6 postoperative day, further testing may not be necessary. In cases where CRP values do not rapidly decrease, a wound infection after spinal surgery must be considered, as clinical signs may be lagging behind. However, other postoperative infectious complications such as urinary tract infections or pneumonia may also cause high CRP values and need to be recognized.

A large prospective randomized trial is necessary to establish whether the time periods we suggest for measurement of CRP are sufficient for early detection of infectious complications after spinal surgery. The same study may answer the question as to how many delayed infectious complications (after day 6) are missed by minimizing CRP-controls as propagated above.
